# Efficacy and safety of add-on mirogabalin to conventional therapy for the treatment of peripheral neuropathic pain after thoracic surgery: the multicenter, randomized, open-label ADMIT-NeP study

**DOI:** 10.1186/s12885-023-11708-2

**Published:** 2024-01-15

**Authors:** Takuro Miyazaki, Keitaro Matsumoto, Toshihiko Sato, Isao Sano, Katsuro Furukawa, Koichiro Shimoyama, Ryotaro Kamohara, Makoto Suzuki, Masamichi Kondou, Norihiko Ikeda, Shunsuke Tabata, Kazuhito Shiosakai, Takeshi Nagayasu, on behalf of the ADMIT-NeP Study group, Ryoichiro Doi, Ryuichi Waseda, Akihiro Nakamura, Keiko Akao, Go Hatachi, Tsutomu Tagawa, Makoto Imai, Koei Ikeda, Masaru Hagiwara

**Affiliations:** 1 Department of Surgical Oncology, Nagasaki University Graduate School of Biomedical Sciences, 1-7-1 Sakamoto, Nagasaki, 852-8501 Japan; 2 Department of Thoracic Surgery, Sasebo City General Hospital, Sasebo, Japan; 3 Department of General Thoracic, Breast, and Pediatric Surgery, Fukuoka University School of Medicine, Fukuoka, Japan; 4 Department of Respiratory Surgery, The Japanese Red Cross Nagasaki Genbaku Hospital, Nagasaki, Japan; 5 Department of Thoracic Surgery, Ehime Prefectural Central Hospital, Matsuyama, Japan; 6 Chest Surgery, National Hospital Organization Nagasaki Medical Center, Omura, Japan; 7 Department of Thoracic Surgery, Oita Prefectural Hospital, Oita, Japan; 8 Department of Thoracic Surgery, Kumamoto University Hospital, Kumamoto, Japan; 9 Department of Thoracic and Breast Surgery, Ureshino Medical Center, Ureshino, Japan; 10 Department of Surgery, Tokyo Medical University, Tokyo, Japan; 11 Primary Medical Science Department, Daiichi Sankyo Co., Ltd, Tokyo, Japan; 12 Data Intelligence Department, Daiichi Sankyo Co., Ltd, Tokyo, Japan

**Keywords:** Chronic postsurgical pain, Mirogabalin, Postoperative neuropathic pain, Post thoracotomy pain syndrome, Thosracic surgery

## Abstract

**Background:**

For chronic pain after thoracic surgery, optimal timing of its diagnosis and effective treatment remains unresolved, although several treatment options are currently available. We examined the efficacy and safety of mirogabalin, in combination with conventional pain therapy (nonsteroidal anti-inflammatory drugs and/or acetaminophen), for treating peripheral neuropathic pain (NeP) after thoracic surgery.

**Methods:**

In this multicenter, randomized, open-label, parallel-group study, patients with peripheral NeP were randomly assigned 1:1 to mirogabalin as add-on to conventional therapy or conventional treatment alone.

**Results:**

Of 131 patients of consent obtained, 128 were randomized (mirogabalin add-on group, 63 patients; conventional treatment group, 65 patients). The least squares mean changes (95% confidence interval [CI]) in Visual Analogue Scale (VAS) score for pain intensity at rest from baseline to Week 8 (primary endpoint) were − 51.3 (− 54.9, − 47.7) mm in the mirogabalin add-on group and − 47.7 (− 51.2, − 44.2) mm in the conventional group (between-group difference: − 3.6 [95% CI: − 8.7, 1.5], *P* = 0.161). However, in patients with Self-administered Leeds Assessment of Neuropathic Symptoms and Signs (S-LANSS) score (used for the screening of NeP) ≥ 12 at baseline, the greater the S-LANSS score at baseline, the greater the decrease in VAS score in the mirogabalin add-on group, while no such trend was observed in the conventional treatment group (post hoc analysis). This between-group difference in trends was statistically significant (interaction *P* value = 0.014). Chronic pain was recorded in 7.9% vs. 16.9% of patients (*P* = 0.171) at Week 12 in the mirogabalin add-on vs. conventional treatment groups, respectively. Regarding activities of daily living (ADL) and quality of life (QOL), changes in Pain Disability Assessment Scale score and the EQ-5D-5L index value from baseline to Week 8 showed significant improvement in the mirogabalin add-on group vs. conventional treatment group (*P* < 0.001). The most common adverse events (AEs) in the mirogabalin add-on group were dizziness (12.7%), somnolence (7.9%), and urticaria (3.2%). Most AEs were mild or moderate in severity.

**Conclusions:**

Addition of mirogabalin to conventional therapy did not result in significant improvement in pain intensity based on VAS scores, but did result in significant improvement in ADL and QOL in patients with peripheral NeP after thoracic surgery.

**Trial registration:**

Japan Registry of Clinical Trials jRCTs071200053 (registered 17/11/2020).

**Supplementary Information:**

The online version contains supplementary material available at 10.1186/s12885-023-11708-2.

## Background

According to the International Association for the Study of Pain, chronic postsurgical pain (CPSP) is defined as pain that continues for ≥ 3 months following a surgical procedure and is not attributable to other causes [1]. CPSP may last many months or even years in some cases [2]. Even mild pain—if it is persistent and chronic in nature—results in prolonged opioid use, impaired physical function, decreased physical and social activities, and increased healthcare costs [3–5]. Thus, the optimal perioperative management of CPSP is one of the top priorities for research in anesthesiology and perioperative pharmacotherapy [6]. The incidence of CPSP varies between 5 and 85% depending on the operational definition and surgical procedure; thoracotomy is associated with a relatively high frequency of CPSP, also known as post thoracotomy pain syndrome [7–10].

Of patients who develop CPSP after thoracic surgery, some patients have a neuropathic pain (NeP) component [11, 12], which has been associated with more marked reduction in physical function and quality of life than CPSP without the neuropathic component [13]. Current treatment options for pain control after thoracic surgery are as follows: in the perioperative period, local anesthetics (epidural anesthesia, paravertebral body block, intercostal nerve block); in the postoperative period, oral anti-inflammatory analgesics (e.g., non-steroidal anti-inflammatory drugs [NSAID] and/or acetaminophen) [14, 15]. Additionally, tricyclic antidepressants, serotonin–noradrenaline reuptake inhibitors (e.g., duloxetine), and gabapentinoids (e.g., gabapentin and pregabalin) are recommended as first-line treatment for NeP [16, 17]. However, clinical outcomes have varied; pregabalin has shown some effectiveness in reducing pain after thoracic surgery but lacked efficacy during the critical early postoperative period [18–20]. Furthermore, recent systematic reviews on pharmacological, perioperative interventions for CPSP reported that minimal progress has been made over the past decade because of inadequate study designs and the low quality of studies [21, 22]. Overall, despite the presence of available treatment, it is clear that for chronic pain after thoracic surgery, optimal timing of its diagnosis and effective treatment remains unresolved.

Mirogabalin besylate (hereinafter referred to as mirogabalin) is an oral gabapentinoid with analgesic effects via binding to the α_2_δ subunit of voltage-gated calcium channels [23]. Mirogabalin has been approved for the treatment of NeP [24]; both peripheral NeP in several Asian countries and central NeP in Japan [25]. The efficacy and safety of mirogabalin have been demonstrated for the treatment of diabetic peripheral NeP [26–28], postherpetic neuralgia [29, 30], and central NeP after spinal cord injury [25] in phase 3 clinical trials and a meta-analysis. Furthermore, a pooled analysis of two phase III clinical trials showed that mirogabalin exhibits a pain relief effect from as early as 2 days after administration [31]. However, evidence of mirogabalin for the treatment of NeP after thoracic surgery is lacking.

The present study aimed to examine the efficacy and safety of mirogabalin, in combination with conventional pain therapy, for the treatment of peripheral NeP after thoracic surgery.

## Methods

### Study design

Details of the study design and protocol have been published previously [32]. The ADd-on MIrogabalin to conventional Therapy for the treatment of peripheral Neuropathic Pain after thoracic surgery (ADMIT-NeP) study was a multicenter, randomized, open-label, parallel-group, interventional study conducted in 14 medical institutions in Japan between December 2020 and September 2022 (Additional file 1). A complete list of investigators and institutions is shown in Additional file 2. The study was conducted in accordance with the Declaration of Helsinki and the Clinical Research Act (promulgated April 14, 2017). In addition, all applicable local, national, and international legislation was applied. The study protocol and associated documentation were approved by the Clinical Research Review Board in Nagasaki University (approval number CRB7180001), and permission to conduct the study was obtained from the administrators of each participating medical institution. This study was registered in the Japan Registry of Clinical Trials under the identifier jRCTs071200053.

Eligible patients were randomly assigned to each treatment group by a registration system using a permuted block method (ratio 1:1). The stratification factors used in this study were a Visual Analogue Scale (VAS) score < 60 mm vs. ≥ 60 mm at baseline and study site.

Administration of the study drugs, set as baseline, was started at 1 or 2 days after the removal of the chest drain. The study did not restrict the use of NSAID or acetaminophen from immediately after surgery to the start of administration of the study drug. In the conventional treatment group, NSAID and/or acetaminophen were prescribed per usual practice and in accordance with the Japanese package insert (including on-demand use) and insurance coverage. Patients were required to maintain a stable treatment regimen during the study. If the given medication did not adequately control pain, the investigator was allowed to increase the dose or to prescribe medications other than the prohibited concomitant medications.

In the mirogabalin add-on group, in addition to conventional treatment, patients received mirogabalin for 8 weeks. The dosage of mirogabalin was adjusted according to the Japanese package insert. Patients with creatinine clearance (CrCL) ≥ 60 mL/min received mirogabalin at 5 mg twice daily (BID) during the first week, 10 mg BID during the second week, and 15 mg BID or 10 mg BID during the third week and onwards. Patients with CrCL ≥ 30 mL/min and < 60 mL/min received mirogabalin 2.5 mg BID during the first week, 5 mg BID during the second week, and 7.5 or 5 mg BID during the third week and onwards.

### Patients

After informed consent (documented by the study investigator) was obtained from patients who had undergone lung resection at the participating medical institutions, patients were screened for study eligibility as previously reported in detail [32]. The key inclusion criteria were as follows: patients aged ≥ 20 years at the time of informed consent who underwent lung resection (for any medical condition) and were enrolled within 1–2 days after removal of the chest drain at the time of lung resection; with a VAS score of ≥ 40 mm (range 0–100 mm), with 0 mm meaning no pain and 100 mm meaning the worst pain imaginable for perioperative pain at rest at the time of enrollment; with hypoesthesia under the intercostal nerve of the intercostal space at the wound site (to identify postoperative pain mainly caused by NeP); and no residual effect of epidural anesthesia at enrollment.

To ensure an accurate and consistent diagnosis of peripheral neuropathy after thoracic surgery, a NeP diagnostic algorithm was used for subjective symptoms that included a questionnaire and a pin-prick sensation test as an objective assessment of symptoms based on a grading system developed by the International Association for the Study of Pain Special Interest Group on Neuropathic Pain [33]. The loss of pin-prick sensation was evaluated at registration as previously described [32]. Neuropathy was also diagnosed based on the presence of hypoesthesia at the surgical wound site including port and drain insertion sites.

The key exclusion criteria included total pleuropulmonary resection or pleurectomy; prior thoracotomy or thoracoscopic surgery resulting in neuropathy that continued until the time of the current surgery; serious liver dysfunction at enrollment; CrCL (Cockcroft–Gault equation) < 30 mL/min within 3 months prior to enrollment; use of NeP medication from 1 month before surgery to the time of enrollment; neoadjuvant chemotherapy within 2 months before surgery; severe pain outside the perioperative wound area complicating the assessment of efficacy in this study; and patients deemed inappropriate for participation in the study by the investigator.

Prohibited concomitant drugs included pregabalin and gabapentin, duloxetine, tramadol, platinum chemotherapy agents, probenecid and cimetidine, and lorazepam. Prohibited concomitant therapies included postoperative nerve block, surgical procedures, or any other intervention (e.g., electrical stimulation, radiation therapy) that could have affected the evaluation of the effectiveness of the study drugs.

### Endpoints

The primary endpoint was the change in pain intensity from baseline to Week 8, measured by VAS at rest. The following secondary endpoints were assessed: the percentage of patients with a Self-administered Leeds Assessment of Neuropathic Symptoms and Signs (S-LANSS), which is an assessment tool for NeP, score of ≥ 12 at Weeks 2, 4, and 8, [34]; the change from baseline to Week 8 in Pain Disability Assessment Scale (PDAS) score for assessment of activities of daily living (ADL) (Week 8) [35]; 5-level EQ-5D (EQ-5D-5L) score for assessment of quality of life (QOL) (Week 8) [36]; the percentage of patients with chronic pain at Weeks 8 and 12 in each treatment group; the percentage of patients with improvements in pain intensity from baseline to Week 8 of ≥ 30% and ≥ 50%, measured using VAS at rest; the change from baseline to Week 8 in pain intensity based on VAS while coughing and VAS for sleep disturbance (Day 1 and Weeks 2, 4, and 8; plus Day 3 at the physician’s discretion); and Patient Global Impression of Change (PGIC) at Week 8 [37]. Chronic pain was judged to occur when a patient met both of the following criteria: having pain related to their chest surgery; and having pain limiting their daily life [38]. The safety endpoint was the occurrence of adverse events (AEs) and adverse drug reactions (ADRs). AEs that occurred after randomization and initiation of the assigned study drug, or that worsened relative to the pre-treatment status were recorded. An ADR was defined as an AE judged by the physician to have a causal relationship with the study drug. Treatment completion rates were assessed, and data on baseline patient, surgical, and treatment characteristics were also collected.

### Sample size

Sample size calculations have been previously described [32]. Briefly, the number of patients needed to ensure 90% power at a two-sided significance level of 5% was 126 (*N* = 63 in each treatment group). Therefore, after accounting for possible dropouts, the target sample size was set at 150 patients (*N* = 75 per group).

### Statistical analyses

For baseline data, categorical variables were summarized as frequency and percentage, and continuous variables were summarized as mean ± standard deviation (SD) and median (interquartile range). The modified intention-to-treat (mITT) population was used for the primary efficacy analyses and was defined as all randomized patients who received at least one dose of the study drug. To calculate the mean differences between groups (mirogabalin add-on group minus conventional treatment group), 95% confidence intervals (CIs), and *P* values for the primary endpoint data, a linear mixed model for repeated measures (MMRM) was used. Detailed methods for the MMRM have been reported previously [32]. Summary statistics were calculated for each time point and change from baseline in each treatment group. For the secondary endpoints, frequency tables or summary statistics were reported using the mITT population.

The per-protocol set was used for sensitivity analyses for efficacy and was defined as all patients in the mITT population who adhered to the study protocol. For the sensitivity of the primary endpoint, detailed analysis methods have been previously reported [32].

The safety analysis set was defined as all patients who were enrolled in the study and received at least one dose of the study drug. AEs were coded using the Japanese Medical Dictionary for Regulatory Activities version 25.0. To calculate the proportion of patients who completed treatment at 8 weeks after thoracic surgery, the number of patients receiving the effective dose at Week 8 was divided by the number of patients at the start of the initial dose (Week 1).

The significance level for hypothesis testing was set at 5% (two-sided), and the CI for both sides was 95%. Statistical analyses were performed using SAS version 9.4 (SAS Institute, Inc., Cary, NC, USA) and Microsoft Excel 2016 (Microsoft Corp., Redmond, WA, USA). Data management and study dissemination has been previously described in detail [32].

## Results

### Patients

**Fig. 1:**
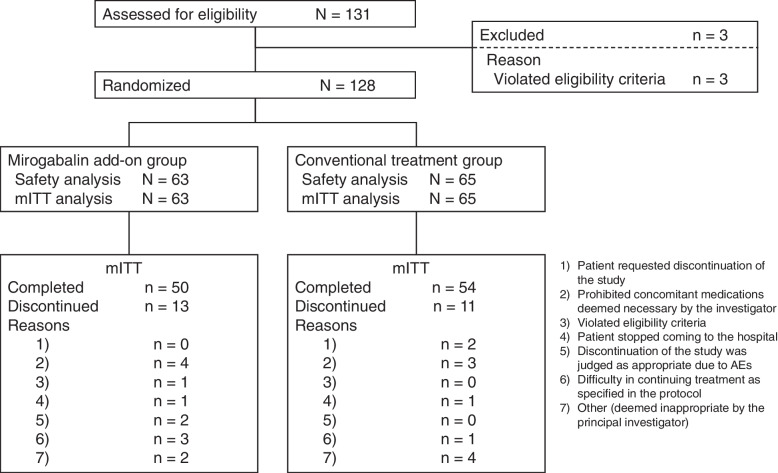
Patient disposition. *AEs, *adverse events; *mITT, *modified intention-to-treat

As it was difficult to recruit patients who met the eligibility criteria during the enrollment period because of the COVID-19 pandemic, enrollment was completed without reaching the target sample size (*N* = 150) despite extending the registration period by 5 months from December 31, 2021 to May 31, 2022. Informed consent was obtained from 131 patients who had undergone lung resection; of these, 128 patients who met the eligibility criteria were enrolled in the study (Fig. 1). Both the mITT population and safety analysis set included 63 patients in the mirogabalin add-on group and 65 patients in the conventional treatment group.

The proportions of patients who completed treatment were 79.4% in the mirogabalin add-on group and 83.1% in the conventional treatment group. The most common reason for study withdrawal was the use of prohibited concomitant medications as deemed necessary by the investigator.

Patient demographic and clinical characteristics for the mITT population are shown in Table 1. In the mirogabalin add-on group and the conventional treatment group, the respective mean ages (67.9 vs. 65.7 years), proportions of female patients (54.0% vs. 61.5%), mean body mass index (22.5 vs. 23.8 kg/m^2^), mean CrCL values (78.0 vs. 84.8 mL/min), proportions of patients with CrCL 30 to < 60 mL/min (30.2% vs. 27.7%), mean VAS score at rest (58.6 vs. 57.9 mm), and proportions of patients with VAS score at rest ≥ 60 (38.1% vs. 38.5%) at enrollment were similar. The most frequent indication of surgery was primary lung cancer (85.7% vs. 75.4%). The most frequent approach method was thoracotomy (44.4%) in the mirogabalin add-on group and video-assisted thoracoscopic surgery (55.4%) in the conventional treatment group. The mean operation time and the duration from lung resection to chest drain removal, duration from chest drain removal to registration, and distribution of blood loss were similar between the two treatment groups. NSAID were prescribed to 69.8% and 76.9% of patients in the mirogabalin and conventional treatment groups, respectively, and the most common type of NSAID prescribed was loxoprofen. Acetaminophen was prescribed to 57.1% and 78.5% patients in the mirogabalin and conventional treatment groups, respectively. Similar results regarding patient demographic and clinical characteristics were obtained in the per-protocol set (data not shown).

**Table 1:** Baseline patient demographic and clinical characteristics (mITT population)

Characteristics	Mirogabalin add-on group (*N* = 63)	Conventional treatment group (*N* = 65)
Age, years	67.9 ± 12.4	65.7 ± 14.1
≥ 65 years	42 (66.7)	41 (63.1)
Sex		
Male	29 (46.0)	25 (38.5)
Female	34 (54.0)	40 (61.5)
BMI, kg/m^2^	22.5 ± 3.3	23.8 ± 4.7
CrCL, mL/min	78.0 ± 28.0	84.8 ± 40.9
≥ 60	44 (69.8)	47 (72.3)
30 to < 60	19 (30.2)	18 (27.7)
VAS score at rest, mm	58.6 ± 13.4	57.9 ± 12.4
40 to < 60	38 (60.3)	40 (61.5)
≥ 60	24 (38.1)	25 (38.5)
Duration from lung resection to chest drain removal, days	2.7 ± 2.1	2.6 ± 1.8
Median (Q1, Q3)	2.0 (1.0, 3.0)	2.0 (2.0, 3.0)
Duration from chest drain removal to registration		
1 day	46 (73.0)	50 (76.9)
2 days	17 (27.0)	15 (23.1)
Duration from lung resection to discharge, days	9.2 ± 4.8	17.2 ± 49.8
Median (Q1, Q3)	8.0 (6.0, 10.0)	7.0 (6.0, 9.0)
Approach method		
Thoracotomy	28 (44.4)	20 (30.8)
VATS	25 (39.7)	36 (55.4)
Robotic	10 (15.9)	9 (13.8)
Use of rib spreader	28 (44.4)	20 (30.8)
Diagnosis at admission		
Primary lung cancer	54 (85.7)	49 (75.4)
Metastatic lung cancer	6 (9.5)	3 (4.6)
Mediastinal tumor	0 (0.0)	2 (3.1)
Pneumothorax	1 (1.6)	1 (1.5)
Other	2 (3.2)	10 (15.4)
Operation time, min	188.5 ± 79.0	189.3 ± 64.8
Median (Q1, Q3)	183.0 (143.0, 221.0)	182.0 (154.0, 229.0)
Blood loss		
≤ 10 mL	30 (47.6)	32 (49.2)
> 10 to ≤ 100 mL	23 (36.5)	25 (38.5)
> 100 to ≤ 200 mL	4 (6.3)	3 (4.6)
> 200 to ≤ 300 mL	1 (1.6)	1 (1.5)
> 300 mL	5 (7.9)	4 (6.2)
Maximum wound size, cm	6.7 ± 4.0	6.5 ± 4.6
Median (Q1, Q3)	6.0 (4.0, 8.0)	5.0 (4.0, 8.0)
^a^NSAID	44 (69.8)	50 (76.9)
^a^Acetaminophen	36 (57.1)	51 (78.5)

Data are mean ± SD or n (%) unless otherwise indicated

*BMI* body mass index, *CrCL* creatinine clearance, *mITT* modified intention-to-treat, *NSAID* non-steroidal anti-inflammatory drugs, *Q* quartile, *SD* standard deviation, *VAS* Visual Analogue Scale, *VATS* video-assisted thoracoscopic surgery

^a^Data are during 8-week from baseline

The daily dose of mirogabalin according to renal function in the mITT population is shown in Additional file 3. Among patients with normal renal function and mild renal impairment (CrCL ≥ 60 mL/min) in the mirogabalin add-on group, 16/42 (38.1%) and 20/42 (47.6%) patients received effective doses of 10 mg BID and 15 mg BID at Week 8, respectively. Among patients with moderate renal impairment (CrCL 30 to < 60 mL/min) in the mirogabalin add-on group, 6/16 (37.5%) and 9/16 (56.3%) patients received effective doses of 5 mg BID and 7.5 mg BID at Week 8, respectively.

### Effect on pain intensity

The least squares (LS) mean changes (95% CI) in VAS score for pain intensity at rest from baseline to Week 8 (primary endpoint) by MMRM analysis were − 51.3 (− 54.9, − 47.7) mm in the mirogabalin add-on group and − 47.7 (− 51.2, − 44.2) mm in the conventional treatment group, respectively (Table 2). The difference between groups in the LS mean change (by MMRM analysis) of the VAS score for pain intensity at rest was − 3.6 mm (95% CI: − 8.7, 1.5), but did not reach statistical significance (*P* = 0.161) compared with the conventional treatment group. A similar tendency was observed in the sensitivity analysis of the per-protocol set (data not shown).

**Table 2:** Change in VAS score at rest from baseline to Week 8 (MMRM analysis; mITT population)

Parameter	Mirogabalin add-on group (*N* = 62)	Conventional treatment group (*N* = 64)
LS mean change (95% CI) from baseline, mm	− 51.3 (− 54.9, − 47.7)	− 47.7 (− 51.2, − 44.2)
Difference in LS mean change (95% CI), mm	− 3.6 (− 8.7, 1.5)	
*P* value^a^	0.161	-

*CI* confidence interval, *LS* least squares, *mITT* modified intention-to-treat, *MMRM* mixed model for repeated measures, *VAS* Visual Analogue Scale

^a^vs. the conventional treatment group

**Fig. 2:**
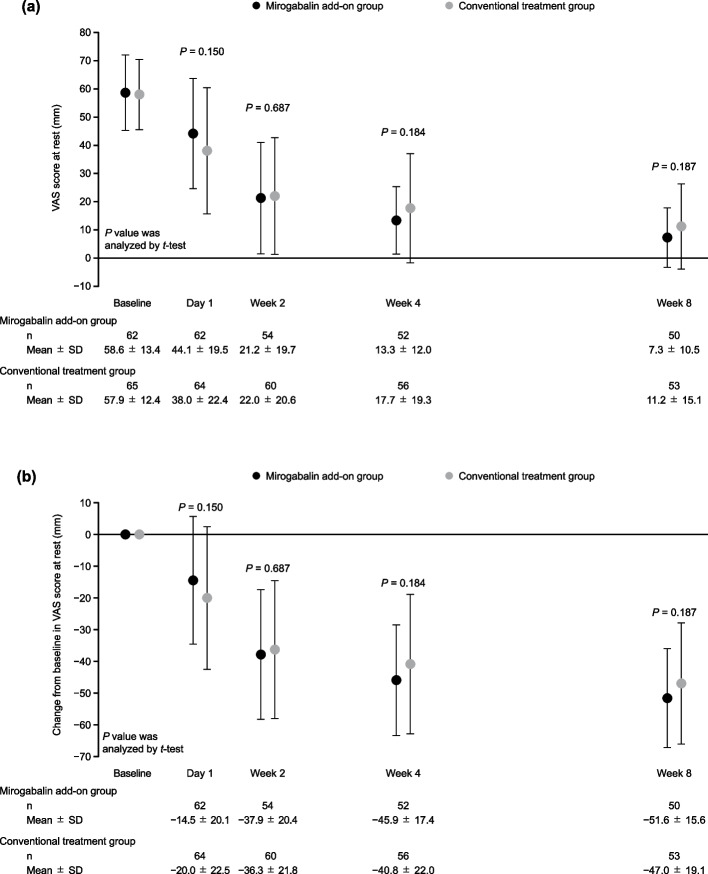
(**a**) VAS score at rest and (**b**) its change from baseline (mITT population). Data are mean ± SD. No statistical tests were conducted for the results shown in (**a**); analysis by* t*-test was conducted to obtain the *P* values for intergroup differences in (**b**). *mITT,* modified intention-to-treat; *SD,* standard deviation; *VAS,* Visual Analogue Scale

**Fig. 3:**
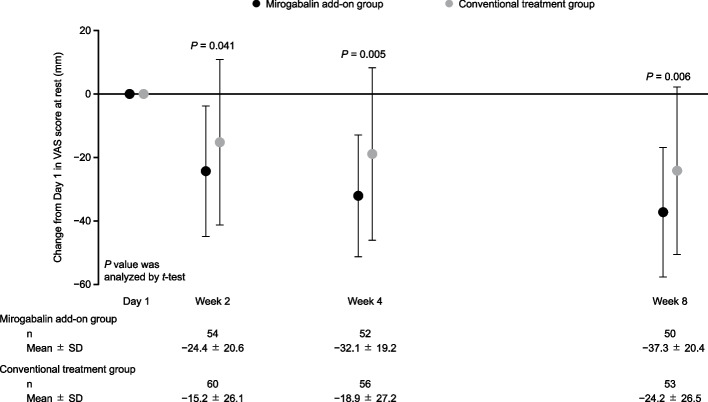
Change from Day 1 in VAS score at rest (mITT population, post hoc analysis). Data are mean ± SD. *P* values for intergroup differences were calculated by *t*-test. *mITT,* modified intention-to-treat; *SD,* standard deviation; *VAS,* Visual Analogue Scale

**Fig. 4:**
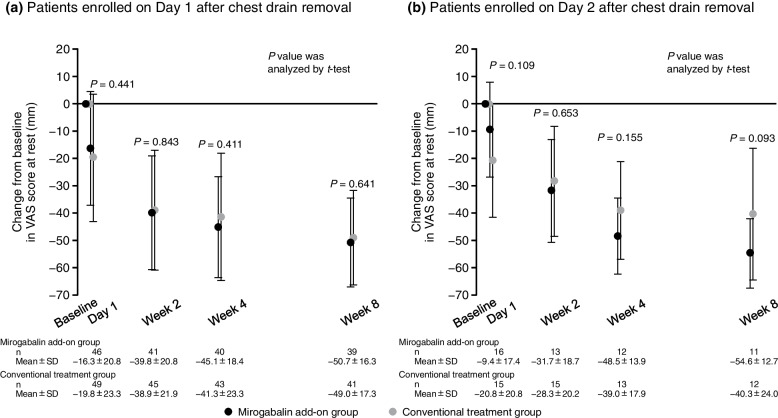
Change from baseline in VAS score at rest by enrollment on Day 1 and Day 2 after chest drain removal (mITT population, post hoc analysis). Duration from lung resection to chest drain removal was 1 day (**a**) or 2 days (**b**). Data are mean ± SD. *P* values for intergroup differences were calculated by *t*-test. *mITT,* modified intention-to-treat; *SD,* standard deviation; *VAS,* Visual Analogue Scale

The VAS score at rest and its change from baseline are shown in Fig. 2. The VAS score at rest decreased during the treatment period in both treatment groups. In particular, from baseline to Day 1, the VAS score decreased rapidly after the start of treatment, suggesting that nociceptive pain may account for a larger proportion of postsurgical pain than NeP. Thus, as a post hoc analysis, we examined 1) the change in VAS score at rest from Day 1 to Weeks 2, 4, and 8, and 2) the change in VAS score at rest from baseline to Day 1 and Weeks 2, 4, and 8 by enrollment on Day 1 and Day 2 after chest drain removal. The reduction in VAS score at rest from Day 1 to Weeks 2, 4, and 8 was significantly greater in the mirogabalin add-on group than in the conventional treatment group (all *P* < 0.05) (Fig. 3). No significant intergroup differences in the change in VAS scores at rest from baseline to Week 8 were observed, regardless of the duration from chest drain removal to enrollment (Fig. 4).

The VAS score for pain intensity while coughing was also improved in both treatment groups, and there was no statistically significant difference between the two treatment groups (Additional file 4).

Both ≥ 30% and ≥ 50% responder rates for the VAS score at rest from baseline to Week 8 were similar in both treatment groups (98.0% vs. 92.5%, *P* = 0.364 for the ≥ 30% responder rates; 94.0% vs. 92.5%, *P* = 1.000 for the ≥ 50% responder rates).

Changes in VAS score at rest from baseline to Week 8 according to the type of lung resection are shown in Additional file 5.

### S-LANSS and pain intensity

At baseline, the percentages of patients with an S-LANSS score ≥ 12 were 50.0% and 41.5% in the mirogabalin add-on group and the conventional treatment group, respectively. The percentage of patients with an S-LANSS score ≥ 12 significantly decreased from baseline (50.0%) to Week 8 (20.0%) in the mirogabalin add-on group (*P* = 0.003), and no statistically significant reduction was observed in the conventional treatment group (baseline, 41.5%; Week 8, 30.2%, *P* = 0.134) (Table 3). There was no statistically significant difference in the percentage of patients with S-LANSS score ≥ 12 at Week 8 between the two treatment groups (*P* = 0.264).

**Table 3:** Patients with S-LANSS score ≥ 12 (mITT population)

	**Mirogabalin add-on group**			**Conventional treatment group**			**Fisher** ***P* value**^**b**^
	**n**	**Patients with S-LANSS score of ≥ 12**	**McNemar** ***P* value**^**a**^	**n**	**Patients with S-LANSS score of ≥ 12**	**McNemar** ***P* value**^**a**^	
Baseline	60	30 (50.0)	-	65	27 (41.5)	-	0.373
Week 2	54	26 (48.1)	1.000	59	27 (45.8)	0.690	0.852
Week 4	52	22 (42.3)	0.690	56	19 (33.9)	0.327	0.430
Week 8	50	10 (20.0)	0.003	53	16 (30.2)	0.134	0.264

Data are *n (%) mITT* modified intention-to-treat, *S-LANSS* Self-administered Leeds Assessment of Neuropathic Symptoms and Signs

^a^vs. baseline

^b^vs. the conventional treatment group

**Fig. 5:**
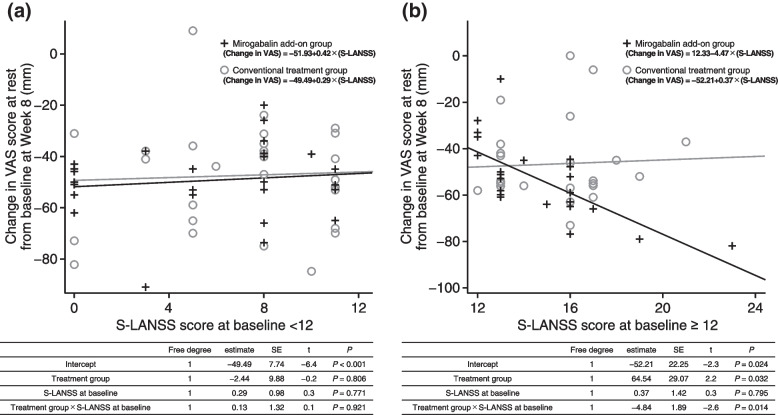
Association between change in VAS score at rest from baseline to Week 8 and baseline S-LANSS score (**a**) < 12 and (**b**) ≥ 12 (post hoc analysis). *SE,* standard error; *S-LANSS,* Self-administered Leeds Assessment of Neuropathic Symptoms and Signs; *VAS,* Visual Analogue Scale

Because it was suspected that nociceptive pain may have a strong influence on the effect of mirogabalin on pain intensity, as mentioned above, we performed a post hoc analysis to examine the associations between the change in VAS score at rest from baseline to Week 8 and baseline S-LANSS score of 12, which was the cut-off value for the identification of NeP [39] (Fig. 5). Degrees of freedom, estimates, standard errors, t values, and* P* values were analyzed by regression analysis with treatment, S-LANSS score at baseline, and interaction between the treatment and S-LANSS score as explanatory variables. In patients with an S-LANSS score of ≥ 12 at baseline, the greater the S-LANSS score at baseline, the greater the decrease in VAS score in the mirogabalin add-on group; no such trend was observed in the conventional treatment group. This difference in trends between the two groups was statistically significant (interaction *P* value = 0.014).

### Effect on chronic pain

The percentages of patients with chronic pain at Weeks 8 and 12 were lower in the mirogabalin add-on group than in the conventional treatment group (at Week 8, 14.3% vs. 26.2%, *P* = 0.113; at Week 12, 7.9% vs. 16.9%, *P* = 0.171), although no statistically significant difference was observed (Table 4).

**Table 4:** Percentages of patients with chronic pain (mITT population)

	**Chronic pain**	**Mirogabalin add-on group** **(*N* = 63)**	**Conventional treatment group** **(*N* = 65)**	**Fisher** ***P* value**^**a**^
Week 8	No	41 (65.1)	35 (53.8)	0.113
	Yes	9 (14.3)	17 (26.2)	
	No test	13 (20.6)	13 (20.0)	
Week 12	No	44 (69.8)	39 (60.0)	0.171
	Yes	5 (7.9)	11 (16.9)	
	No test	14 (22.2)	15 (23.1)	

Data are *n (%) mITT* modified intention-to-treat

^a^vs. the conventional treatment group

### Effect on ADL and QOL

Both PDAS and EQ-5D-5L scores significantly improved from baseline to Week 8 in both treatment groups (all *P* < 0.001) (Table 5); however, these changes from baseline to Week 8 were significantly greater in the mirogabalin add-on group than in the conventional treatment group (PDAS score, − 24.1 ± 14.1 vs. − 14.4 ± 14.8,* P* < 0.001; EQ-5D-5L score, 0.3363 ± 0.2127 vs. 0.1798 ± 0.1922, *P* < 0.001).

**Table 5:** Change in PDAS and EQ-5D-5L scores from baseline to Week 8 (mITT population)

Parameter	Mirogabalin add-on group (*N* = 63)	Conventional treatment group (*N* = 65)
**PDAS score**		
**Baseline, n**	60	64
Mean ± SD	32.3 ± 14.2	28.9 ± 14.2
Median (Q1, Q3)	32.5 (22.0, 44.0)	27.5 (20.0, 40.0)
Min, Max	0, 57	0, 60
**Week 8, n**	50	53
Mean ± SD	8.7 ± 9.6	11.6 ± 11.1
Median (Q1, Q3)	4.5 (2.0, 13.0)	9.0 (2.0, 19.0)
Min, Max	0, 34	0, 38
Change from baseline	− 24.1 ± 14.1	− 14.4 ± 14.8
*P* value^a^	< 0.001	< 0.001
*P* value^b^	< 0.001	-
**EQ-5D-5L score, index value**		
**Baseline, n**	60	65
Mean ± SD	0.5179 ± 0.2095	0.6153 ± 0.2188
Median (Q1, Q3)	0.5164 (0.3898, 0.6528)	0.6392 (0.5187, 0.7791)
Min, Max	− 0.025, 1.000	− 0.025, 1.000
**Week 8, n**	50	53
Mean ± SD	0.8497 ± 0.1335	0.8385 ± 0.1386
Median (Q1, Q3)	0.8819 (0.7803, 1.0000)	0.8441 (0.7723, 0.8945)
Min, Max	0.438, 1.000	0.455, 1.000
Change from baseline	0.3363 ± 0.2127	0.1798 ± 0.1922
*P* value^a^	< 0.001	< 0.001
*P* value^b^	< 0.001	-

*EQ-5D-5L* 5-level EQ-5D, *mITT* modified intention-to-treat, *PDAS* Pain Disability Assessment Scale, *Q* quartile, *SD* standard deviation

^a^vs. baseline by *t*-test

^b^vs. the conventional treatment group by *t*-test

VAS for sleep disturbance decreased in both treatment groups after starting treatment from baseline to Week 8 (Additional file 6), but intergroup significant differences were not observed during those 8 weeks.

At Week 8, the proportions of patients with PGIC score ≤ 2 (the sum of much and very much improved) were 88.0% and 73.6% in the mirogabalin and conventional treatment groups, respectively (between-group comparison, *P* = 0.083) (Additional file 7).

### Safety

AEs and ADRs occurring in ≥ 2% patients are shown in Table 6. The overall incidence of AEs was 38.1% and 12.3% in the mirogabalin and conventional treatment groups, respectively, and that of ADRs was 23.8% and 0.0%, respectively. The proportion of patients who discontinued treatment because of an AE or ADR was 7.9% or 4.8%, respectively, in the mirogabalin add-on group. No patients in the conventional treatment group discontinued treatment because of an AE or ADR. The most common AEs in the mirogabalin add-on group were dizziness (12.7%), somnolence (7.9%), and urticaria (3.2%). Most AEs were mild or moderate in severity, and no serious ADRs or deaths were reported in either group. The most common AE leading to treatment discontinuation in the mirogabalin add-on group was urticaria (*n* = 2, 3.2%).

**Table 6:** AEs and ADRs occurring in ≥ 2% of patients (safety analysis set)

	**Mirogabalin add-on group** **(*N* = 63)**	**Conventional treatment group** **(*N* = 65)**
**AEs**		
Overall AEs	24 (38.1)	8 (12.3)
Dizziness	8 (12.7)	0 (0.0)
Somnolence	5 (7.9)	0 (0.0)
Urticaria	2 (3.2)	0 (0.0)
Serious AEs	5 (7.9)	3 (4.6)
Discontinuation due to AEs	5 (7.9)	0 (0.0)
Urticaria	2 (3.2)	0 (0.0)
**ADRs**		
Overall ADRs	15 (23.8)	0 (0.0)
Dizziness	8 (12.7)	0 (0.0)
Somnolence	5 (7.9)	0 (0.0)
Serious ADRs	0 (0.0)	0 (0.0)
Discontinuation due to ADRs	3 (4.8)	0 (0.0)

Data are n (%)

Coded using the MedDRA/J, version 25.0

*ADRs* adverse drug reactions, *AEs* adverse events, *MedDRA/J* Japanese Medical Dictionary for Regulatory Activities

## Discussion

The ADMIT-NeP study is the first clinical study to assess the efficacy of 8-week treatment with mirogabalin for pain relief and improvement of ADL and QOL and its safety in patients with peripheral NeP after thoracic surgery. Mirogabalin added on to NSAID and/or acetaminophen did not show statistical significance compared with the conventional treatment for the primary endpoint (change in VAS score for pain intensity at rest from baseline to Week 8); however, there was nominal statistical significance in favor of mirogabalin in several secondary endpoints. In the mirogabalin add-on group, there were significant improvements in ADL and QOL based on the PDAS and EQ-5D-5L compared with the conventional treatment group. Although other efficacy outcomes (the VAS for pain while coughing, NeP based on the S-LANSS score, VAS for sleep disturbance, and PGIC scores) were improved in the mirogabalin add-on group compared with the conventional treatment group, there was no statistically significant difference between the groups. Regarding safety, mirogabalin as add-on to NSAID and/or acetaminophen was generally well tolerated and did not raise any new safety concerns, and most AEs were mild or moderate.

Many previous studies have reported on the efficacy and safety of conventional treatment with duloxetine [40, 41], gabapentin [42–44], and pregabalin [18–20, 45, 46] in patients with postoperative pain. Contrary to what was expected, the present study could not show a statistically significant improvement regarding efficacy outcomes in the mirogabalin add-on group vs. the conventional treatment group. Similarly, some studies have reported the non-superiority of gabapentin and pregabalin vs. control for improving pain in patients after undergoing thoracic surgery [20, 44]. One possible explanation for these results is thought to be a strong pain-improving effect by NSAID and/or acetaminophen. The improvement of VAS for pain intensity at Week 2 in the conventional treatment group of the present study was higher vs. that in previous studies: at rest, − 36.3 vs. − 10.1, and while coughing, − 33.2 vs. − 26.8 [19]; at rest, − 47.0 at Week 8 vs. about − 20 at Day 60 [42]. The stronger pain-improving effect of NSAID and/or acetaminophen in the present study suggests the possibility of a spontaneous healing effect. In this study, the NeP possibly due to intercostal nerve damage may not have been as persistent and severe as diabetic peripheral NeP [26–28], postherpetic neuralgia [29, 30], and central NeP after spinal cord injury [25], which have been examined in previous phase 3 clinical trials of mirogabalin, resulting in the possibility that some patients may have spontaneously recovered. In a previous study of pregabalin [19], even though the pain medication was terminated at Week 2, followed by a 10-week follow-up period during which, in principle, pain medication was not administered, the VAS improved gradually over time during the follow-up period. Another possible explanation is the influence of nociceptive pain. In the present study, a NeP diagnostic algorithm [33] and a test for loss of pin-prick sensation [32] were used to identify patients with peripheral neuropathy while ruling out nociceptive pain after thoracic surgery. However, more than half of patients had an S-LANSS score < 12 at baseline, suggesting that half of patients may have had fewer NeP components. Furthermore, in the present study, a marked decrease in VAS score for pain intensity was observed in the early treatment period (from baseline to Day 1), which reiterates that nociceptive pain might account for a larger proportion of postsurgical pain than NeP. This is also supported by the findings that VAS scores for pain intensity from Day 1 to Week 8 were significantly improved in the mirogabalin add-on group; the intergroup difference in VAS score tended to be greater when the duration from lung resection to chest drain removal was 2 days compared with 1 day. Finally, we examined the relationships between the change in VAS score and baseline S-LANSS score. In the mirogabalin add-on group, the reduction in VAS score at rest from baseline to Week 8 became greater with the higher baseline S-LANSS score, whereas this trend was not observed in the conventional treatment group; these differences in trends between the two groups were statistically significant. Such differences were not observed in patients with S-LANSS score < 12 at baseline. Thus, this study suggests that the addition of mirogabalin to NSAID and/or acetaminophen may have had an additional effect in improving NeP after thoracic surgery in patients who have many NeP components. Further study designed to exclude the influence of nociceptive pain is required.

It is important to reduce not only acute pain but also to prevent the transition to chronic pain. A previous study reported that higher levels of immediate postoperative pain were associated with post thoracotomy pain syndrome [47], and pain management in the immediate early post-operative period is important for reducing the transition to chronic pain. In the present study, the mirogabalin add-on group tended to have lower percentages of patients with S-LANSS score ≥ 12 and chronic pain compared with the conventional treatment group, suggesting that mirogabalin may have inhibited NeP and prevented the transition to chronic pain. Although it is necessary to consider the target population for treatment, early initiation of mirogabalin treatment after thoracic surgery may have clinical benefit.

The goal of treating NeP includes improvement in ADL and QOL, rather than just eliminating the pain [48]. In the present study, the PDAS for assessment of ADL and EQ-5D-5L for assessment of QOL significantly improved in the mirogabalin add-on group compared with the conventional treatment group. Additionally, other QOL indexes, VAS for sleep disturbance and PGIC, improved from baseline to Week 8 in the mirogabalin add-on group, but there were no statistically significant differences compared with the conventional treatment group. These results of VAS for sleep disturbance and PGIC were similar to those regarding pain relief, which may also be attributed to the significant pain-improving effect by NSAID and/or acetaminophen. Considering the significant improvement in PDAS and EQ-5D-5L scores, these results suggest that mirogabalin not only reduces postoperative pain, but also improves ADL and QOL in patients with NeP after thoracic surgery. Other clinical studies of mirogabalin have also reported an improvement in QOL with mirogabalin vs. a control group [49], although the diseases and duration of treatment are different from those of the present study. The VAS for pain intensity is a simple endpoint, but ADL and QOL are integrative endpoints consisting of multiple factors, which may be why a significant effect of mirogabalin could be detected in ADL and QOL.

The incidence of AEs and ADRs was higher in the mirogabalin add-on group vs. the conventional treatment group. In the present study, the major types of AEs were dizziness and somnolence, which were not new and were broadly consistent with those observed in previous trials of mirogabalin in patients with diabetic peripheral NeP and postherpetic neuralgia [27, 30] and other gabapentinoids in patients with thoracotomy [18, 19, 44, 46, 50]. Previous phase III trials of mirogabalin have also reported that weight gain (4.0%–5.0%) and peripheral edema (4.1%–5.3%) were major types of AEs [27, 30], but these did not occur in this study. Although the reason for this is unknown, it has been previously reported that the onset of edema, peripheral edema, and increased weight occurred at a later time between Week 4 and Week 12 of treatment with mirogabalin [31].

This study has some limitations, including those inherent to the open-label design. Therefore, there is the possibility of conscious or unconscious bias, which could have influenced the patients’ responses to the study drug or the patients’ or physicians’ evaluations of efficacy. In the present study, approximately 20% of patients in both groups failed to complete the study. The study was designed assuming a discontinuation rate of 15%, and the discrepancy between this value and the actual results is small. Although the concomitant use of prohibited drugs was the most frequent reason for discontinuation in this study, most cases were discontinued when the prohibited drugs were administered, and thus the effect on the obtained data is considered to be negligible. Excluding these discontinuations, the discontinuation rate is similar to that in previous studies examining the effect of pregabalin on postoperative pain (8%–10.8%) [11, 20, 51], although the duration of the studies and patient characteristics differ. In addition, pain is a subjective symptom, and its assessment is complicated when multiple pain components such as neuropathic and nociceptive pain are present. Although this study attempted to include patients having NeP and no/little nociceptive pain by S-LANSS and guideline-based screening, the simple assessment methods such as VAS used in the primary endpoint might not have accurately assessed NeP. For patients after thoracic surgery, an assessment tool to more accurately evaluate NeP is needed. Although there was no bias in baseline VAS score between the two groups, the mirogabalin group had a higher rate of thoracotomy, which may have influenced the results. This study did not collect information on the number of patients with concomitant use of NSAID and acetaminophen and their doses during the treatment period. These limitations may have influenced the efficacy results and may be one reason why no between-group differences were obtained. The target sample size was not reached because of the impact of the COVID-19 pandemic, and the statistical power of detection was reduced. Because of the relatively short evaluation period of this study, the long-term efficacy and safety of mirogabalin are unknown.

## Conclusions

In the present study, while the concomitant use of mirogabalin and conventional pain relief therapy could not confirm a further significant improvement in pain intensity based on the VAS score, it did elicit significant improvements in ADL and QOL. Moreover, the combination of mirogabalin and the conventional therapy was generally well tolerated. Further studies are needed to clarify the pain-improving effect of mirogabalin in patients with NeP after thoracic surgery, especially by including patients with more NeP components and less nociceptive pain.

### Supplementary Information

**Additional file 1. **Study design.

**Additional file 2. **List of participating institutions and principal investigators.

**Additional file 3. **Mirogabalin daily dose for 12 weeks by renal function at enrollment (mITT population, *N* = 63).

**Additional file 4. **Change in VAS score while coughing from baseline to Week 8 (secondary endpoint) (mITT population).

**Additional file 5. **Changes in VAS score at rest from baseline to Week 8 by type of lung resection in the mITT population.

**Additional file 6. **Change from baseline to Week 8 in VAS score for sleep disturbance (mITT population).

**Additional file 7. **PGIC at Week 8.

## Data Availability

The deidentified participant data and the study protocol will be shared on a request basis for up to 36 months after the publication of this article. Researchers who make the request should include a methodologically sound proposal on how the data will be used; the proposal may be reviewed by the responsible personnel at Daiichi Sankyo Co. Ltd., and the data requestors will need to sign a data access agreement. Please directly contact the corresponding author to request data sharing.

## Abbreviations

ADL: Activities of daily living
ADR: Adverse drug reaction
AE: Adverse event
BID: Twice daily
CI: Confidence interval
CrCL: Creatinine clearance
CPSP: Chronic postsurgical pain
EQ-5D-5L: 5-Level EQ-5D
LS: Least squares
mITT: Modified intention-to-treat
MMRM: Mixed model for repeated measures
NeP: Neuropathic pain
NSAID: Non-steroidal anti-inflammatory drugs
PDAS: Pain Disability Assessment Scale
PGIC: Patient Global Impression of Change
QOL: Quality of life
SD: Standard deviation
SE: Standard error
S-LANSS: Self-administered Leeds Assessment of Neuropathic Symptoms and Signs
VAS: Visual Analogue Scale
VATS: Video-Assisted Thoracoscopic Surgery
