# Supplementary material for: Efficacy and safety of add-on mirogabalin to conventional therapy for the treatment of peripheral neuropathic pain after thoracic surgery: the multicenter, randomized, open-label ADMIT-NeP study
Source: BMC Cancer. 2024 Jan 15;24:80. doi: 10.1186/s12885-023-11708-2 (PMC10788972; doi:10.1186/s12885-023-11708-2)
Supplement: Supplementary file 1 — Additional file 1. Study design. [file 12885_2023_11708_MOESM1_ESM.pdf]

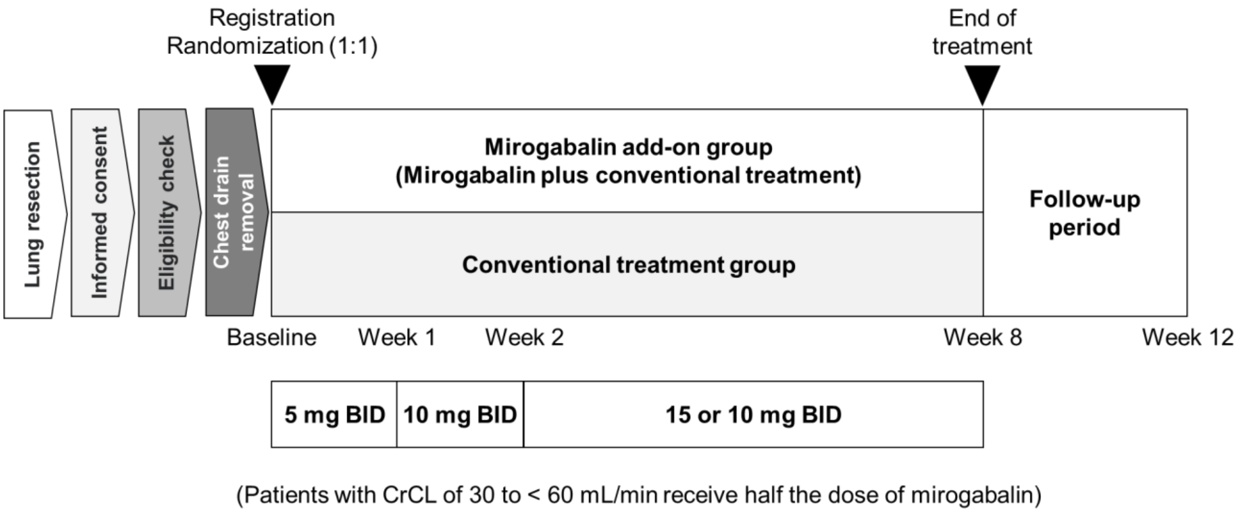

Additional file 1 Study design

The administration of study drugs was started at 1 or 2 days after the removal of the chest drain, which was set as baseline.

BID, twice daily; CrCL, creatinine clearance.
