# Supplementary material for: Efficacy and safety of add-on mirogabalin to conventional therapy for the treatment of peripheral neuropathic pain after thoracic surgery: the multicenter, randomized, open-label ADMIT-NeP study
Source: BMC Cancer. 2024 Jan 15;24:80. doi: 10.1186/s12885-023-11708-2 (PMC10788972; doi:10.1186/s12885-023-11708-2)
Supplement: Supplementary file 2 — Additional file 2. List of participating institutions and principal investigators. [file 12885_2023_11708_MOESM2_ESM.pdf]

Additional file 2 List of participating institutions and principal investigators

Name of institution	Name of the principal investigator at the study site
Nagasaki University Hospital	Takeshi Nagayasu[a]
National Hospital Organization Nagasaki Medical Center	Tsutomu Tagawa
Local incorporated administrative agency Sasebo City General Hospital	Akihiro Nakamura<br>Shigeyuki Morino
National Hospital Organization Ureshino Medical Center	Masamichi Kondou
Ehime Prefectural Central Hospital	Katsuro Furukawa
Kumamoto University Hospital	Makoto Suzuki
University of Miyazaki Hospital	Takanori Ayabe<br>Masaki Tomita
Kagoshima University Graduate School of Medical and Dental Sciences	Masami Sato<br>Kazuhiro Ueda
Oita Prefectural Hospital	Ryotaro Kamohara
Japanese Red Cross Nagasaki Genbaku Hospital	Isao Sano
Niigata University Medical and Dental Hospital	Masanori Tsuchida
University of Tsukuba Hospital	Yukinobu Goto
Tokyo Medical University Hospital	Norihiko Ikeda
Fukuoka University Hospital	Toshihiko Sato

[a] Principal investigator.
