# Supplementary material for: Efficacy and safety of add-on mirogabalin to conventional therapy for the treatment of peripheral neuropathic pain after thoracic surgery: the multicenter, randomized, open-label ADMIT-NeP study
Source: BMC Cancer. 2024 Jan 15;24:80. doi: 10.1186/s12885-023-11708-2 (PMC10788972; doi:10.1186/s12885-023-11708-2)
Supplement: Supplementary file 3 — Additional file 3. Mirogabalin daily dose for 12 weeks by renal function at enrollment (mITT population, N = 63). [file 12885_2023_11708_MOESM3_ESM.pdf]

Additional file 3 Mirogabalin daily dose for 12 weeks by renal function at enrollment (mITT population, N = 63)

**Mirogabalin dose**	**CrCL ≥ 60 mL/min**					**CrCL 30 to < 60 mL/min**				
	**Baseline**	**Day 1**	**Week 2**	**Week 4**	**Week 8**	**Baseline**	**Day 1**	**Week 2**	**Week 4**	**Week 8**
15 mg BID	0 (0.0)	0 (0.0)	15 (34.9)	21 (50.0)	20 (47.6)	0 (0.0)	0 (0.0)	0 (0.0)	0 (0.0)	0 (0.0)
10 mg BID	1 (2.3)	1 (2.3)	22 (51.2)	14 (33.3)	16 (38.1)	0 (0.0)	0 (0.0)	0 (0.0)	0 (0.0)	0 (0.0)
7.5 mg BID	0 (0.0)	0 (0.0)	0 (0.0)	0 (0.0)	0 (0.0)	0 (0.0)	0 (0.0)	7 (43.8)	9 (56.3)	9 (56.3)
5 mg BID	43 (97.7)	43 (97.7)	6 (14.0)	6 (14.3)	6 (14.3)	1 (5.3)	1 (5.3)	8 (50.0)	6 (37.5)	6 (37.5)
2.5 mg BID	0 (0.0)	0 (0.0)	0 (0.0)	0 (0.0)	0 (0.0)	18 (94.7)	18 (94.7)	1 (6.3)	1 (6.3)	1 (6.3)
Other	0 (0.0)	0 (0.0)	0 (0.0)	1 (2.4)	0 (0.0)	0 (0.0)	0 (0.0)	0 (0.0)	0 (0.0)	0 (0.0)

Data are n (%).

BID, twice daily; CrCL, creatinine clearance; mITT, modified intention-to-treat.
