# Supplementary material for: Efficacy and safety of add-on mirogabalin to conventional therapy for the treatment of peripheral neuropathic pain after thoracic surgery: the multicenter, randomized, open-label ADMIT-NeP study
Source: BMC Cancer. 2024 Jan 15;24:80. doi: 10.1186/s12885-023-11708-2 (PMC10788972; doi:10.1186/s12885-023-11708-2)
Supplement: Supplementary file 4 — Additional file 4. Change in VAS score while coughing from baseline to Week 8 (secondary endpoint) (mITT population). [file 12885_2023_11708_MOESM4_ESM.pdf]

**Additional file 4** Change in VAS score while coughing from baseline to Week 8 (secondary endpoint) (mITT population)

| | **Mirogabalin add-on group (N = 63)** | | **Conventional treatment group (N = 65)** | | **Intergroup difference[a]** |
|---|---|---|---|---|---|
| | **Mean ± SD** | **Change from baseline** | **Mean ± SD** | **Change from baseline** | |
| Baseline | 68.3 ± 18.9 | | 66.4 ± 18.8 | | |
| Day 1 | 62.7 ± 20.4 | −5.6 ± 16.2<br>$P = 0.008$[b] | 54.9 ± 23.2 | −11.4 ± 21.1<br>$P < 0.001$[b] | $P = 0.088$ |
| Week 2 | 32.0 ± 21.4 | −36.6 ± 21.2<br>$P < 0.001$[b] | 33.0 ± 25.4 | −33.2 ± 28.2<br>$P < 0.001$[b] | $P = 0.470$ |
| Week 4 | 15.6 ± 13.7 | −53.0 ± 21.0<br>$P < 0.001$[b] | 20.5 ± 23.4 | −44.8 ± 26.2<br>$P < 0.001$[b] | $P = 0.078$ |
| Week 8 | 8.5 ± 12.2 | −59.2 ± 20.0<br>$P < 0.001$[b] | 13.1 ± 19.6 | −51.9 ± 26.7<br>$P < 0.001$[b] | $P = 0.121$ |

[a] vs. the conventional treatment group by *t*-test.

[b] vs. baseline by paired *t*-test.

mITT, modified intention-to-treat; SD, standard deviation; VAS, Visual Analogue Scale.
