# Supplementary material for: Efficacy and safety of add-on mirogabalin to conventional therapy for the treatment of peripheral neuropathic pain after thoracic surgery: the multicenter, randomized, open-label ADMIT-NeP study
Source: BMC Cancer. 2024 Jan 15;24:80. doi: 10.1186/s12885-023-11708-2 (PMC10788972; doi:10.1186/s12885-023-11708-2)
Supplement: Supplementary file 5 — Additional file 5. Changes in VAS score at rest from baseline to Week 8 by type of lung resection in the mITT population. [file 12885_2023_11708_MOESM5_ESM.pdf]

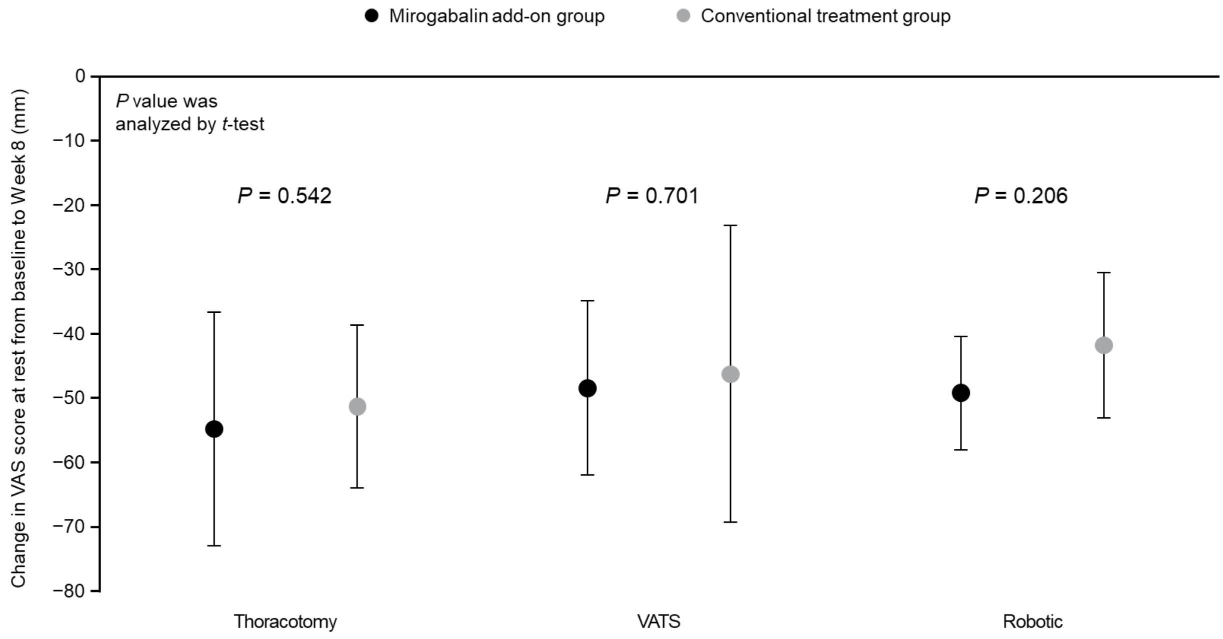

	Thoracotomy		VATS		Robotic	
n	23	14	21	30	6	9
Mean ± SD	−54.9 ± 18.2	−51.5 ± 12.7	−48.6 ± 13.6	−46.4 ± 23.1	−49.3 ± 8.9	−42.0 ± 11.3

Additional file 5 Changes in VAS score at rest from baseline to Week 8 by type of lung resection in the mITT population

Data are mean ± SD. *P* values for intergroup differences were calculated by *t*-test.

mITT, modified intention-to-treat; SD, standard deviation; VAS, Visual Analogue Scale; VATS, video-assisted thoracoscopic surgery.
