# Supplementary material for: Efficacy and safety of add-on mirogabalin to conventional therapy for the treatment of peripheral neuropathic pain after thoracic surgery: the multicenter, randomized, open-label ADMIT-NeP study
Source: BMC Cancer. 2024 Jan 15;24:80. doi: 10.1186/s12885-023-11708-2 (PMC10788972; doi:10.1186/s12885-023-11708-2)
Supplement: Supplementary file 6 — Additional file 6. Change from baseline to Week 8 in VAS score for sleep disturbance (mITT population). [file 12885_2023_11708_MOESM6_ESM.pdf]

Additional file 6 Change from baseline to Week 8 in VAS score for sleep disturbance (mITT population)

	**Mirogabalin add-on group (N = 63)**		**Conventional treatment group (N = 65)**		**Intergroup difference[a]**
	**Mean ± SD**	**Change from baseline**	**Mean ± SD**	**Change from baseline**	
Baseline	46.4 ± 29.6		46.8 ± 31.6		
Day 1	41.1 ± 29.6	−5.3 ± 23.9<br>$P = 0.086$[b]	31.6 ± 24.7	−14.6 ± 33.0<br>$P < 0.001$[b]	$P = 0.074$
Week 2	16.3 ± 19.7	−31.9 ± 28.3<br>$P < 0.001$[b]	21.8 ± 24.9	−24.0 ± 37.3<br>$P < 0.001$[b]	$P = 0.211$
Week 4	8.8 ± 13.1	−39.9 ± 28.9<br>$P < 0.001$[b]	15.4 ± 19.8	−30.2 ± 28.3<br>$P < 0.001$[b]	$P = 0.081$
Week 8	5.6 ± 10.0	−41.4 ± 28.4<br>$P < 0.001$[b]	8.0 ± 12.0	−37.1 ± 32.3<br>$P < 0.001$[b]	$P = 0.474$

[a] vs. the conventional treatment group by *t*-test.

[b] vs. baseline by paired *t*-test.

mITT, modified intention-to-treat; SD, standard deviation; VAS, Visual Analogue Scale.
