# Supplementary material for: Efficacy and safety of add-on mirogabalin to conventional therapy for the treatment of peripheral neuropathic pain after thoracic surgery: the multicenter, randomized, open-label ADMIT-NeP study
Source: BMC Cancer. 2024 Jan 15;24:80. doi: 10.1186/s12885-023-11708-2 (PMC10788972; doi:10.1186/s12885-023-11708-2)
Supplement: Supplementary file 7 — Additional file 7. PGIC at Week 8. [file 12885_2023_11708_MOESM7_ESM.pdf]

Additional file 7 PGIC at Week 8

	mITT population		
	**Mirogabalin add-on group (N = 63)**	**Conventional treatment group (N = 65)**	***P* value**
PGIC evaluation			
No	13 (20.6)	12 (18.5)	0.825[a]
Yes	50 (79.4)	53 (81.5)	
1. Very much improved	6 (12.0)	5 (9.4)	0.596[b]
2. Much improved	38 (76.0)	34 (64.2)	
3. Minimally improved	1 (2.0)	9 (17.0)	
4. No change	3 (6.0)	4 (7.5)	
5. Minimally worse	0 (0.0)	1 (1.9)	
6. Much worse	2 (4.0)	0 (0.0)	
7. Very much worse	0 (0.0)	0 (0.0)	
PGIC (score ≤ 2)	44 (88.0)	39 (73.6)	0.083[a]
PGIC (score ≤ 3)	45 (90.0)	48 (90.6)	1.000[a]

Data are n (%) unless otherwise indicated.

[a] Fisher test.

[b] Cochran–Armitage test.

mITT, modified intention-to-treat; PGIC, Patient Global Impression of Change.
